# Molecular organization, localization and orientation of antifungal antibiotic amphotericin B in a single lipid bilayer

**DOI:** 10.1038/srep32780

**Published:** 2016-09-13

**Authors:** Wojciech Grudzinski, Joanna Sagan, Renata Welc, Rafal Luchowski, Wieslaw I. Gruszecki

**Affiliations:** 1 Department of Biophysics, Institute of Physics, Maria Curie-Skłodowska University, Lublin, Poland

## Abstract

Amphotericin B is a popular antifungal antibiotic, a gold standard in treatment of systemic mycotic infections, due to its high effectiveness. On the other hand, applicability of the drug is limited by its considerable toxicity to patients. Biomembranes are a primary target of physiological activity of amphotericin B and both the pharmacologically desired and toxic side effects of the drug relay on its molecular organization in the lipid phase. In the present work, molecular organization, localization and orientation of amphotericin B, in a single lipid bilayer system, was analysed simultaneously, thanks to application of a confocal fluorescence lifetime imaging microscopy of giant unilamellar vesicles. The results show that the presence of sterols, in the lipid phase, promotes formation of supramolecular structures of amphotericin B and their penetration into the membrane hydrophobic core. The fact that such an effect is substantially less pronounced in the case of cholesterol than ergosterol, the sterol of fungal membranes, provides molecular insight into the selectivity of the drug.

Owing to a dramatic increase in systemic fungal infections, in particular associated with the HIV pandemic and organ transplantation, effective treatment of fungal infections is an important and topical issue^1^,^2^. For several decades amphotericin B (AmB) has been used to treat life-threatening, systemic mycotic infections (see Supplementary information Fig. S1 for a chemical structure). The drug is a gold standard in therapy of fungal infections, due to its high pharmacological effectiveness, despite severe side effects^3^. Activity of numerous laboratories around the world is focused on understanding molecular mechanisms responsible for biological activity of AmB, which could provide indications in elaboration of effective anti-mycotic pharmacological formula of the drug and to design new derivatives of the parent molecule with minimized toxic side effects to patients. Toxicity of AmB is linked to interaction of the drug with physiologically relevant biomolecules, including proteins and membrane-forming lipids^4^. As regarding biomembranes, according to a general understanding, both the therapeutic and toxic side effects are associated with molecular organization of the drug. Several molecular mechanisms have been proposed to be directly associated with the physiological activity of AmB in biomembranes. Among these mechanisms the most popular is formation of pores, independently in two lipid monolayers of a bilayer, assembly of which may yield transmembrane channels^5^. Such pores may be formed with participation of sterols^6^,^7^ or with AmB alone^8^ and can act as transmembrane ion channels affecting ionic equilibrium of a living cell. It has also has been proposed that the presence of AmB molecules in a lipid bilayer environment creates a structural mismatch able to affect structural and dynamic properties of the membrane to a level that facilitates nonspecific, transmembrane ion leakage^9^. Recently, a different molecular mechanism has been proposed, of AmB with respect to biomembranes, which consists in sequestration of sterol molecules from the lipid phase and formation of two-component, AmB-sterol extramembraneous sponge-like structures^10^. In the present work we readdress the problem of molecular organization, localization and orientation of AmB molecules with respect to sterol-free and sterol-containing lipid membranes, with application of very well defined experimental system consisting of a single lipid bilayer and with application of ultrasensitive confocal fluorescence lifetime imaging microscopy (FLIM) technique.

A lipid bilayer is a basic structural scaffold of biomembranes and therefore interaction, localization and orientation of physiologically relevant molecules, including pharmaceuticals, within lipid membranes is an interesting and highly important issue. Orientation of molecules in lipid bilayers can be determined by means of linear dichroism measurements applied to the planar lipid membrane systems^11^,^12^,^13^,^14^,^15^,^16^,^17^. Experiments addressing this issue can be performed in a single lipid bilayer model but in most cases they were conducted with the use of the lipid multi-bilayer systems, owing to the requirement of high signal to noise ratio^13^,^14^,^15^,^17^,^18^. On the other hand, molecules of interest, including AmB, may potentially occupy sites in the intermembrane space of the lipid multi-bilayer system, in addition to locations specific to lipid bilayers: in the headgroup or the membrane core regions. Such a risk makes the multi-bilayer system more doubtful as compared to a single lipid bilayer^19^. Here we apply a precise approach of determination of orientation of molecules with respect to lipid bilayer membranes, based on confocal fluorescence microscopy and imaging of a single unilamellar liposome^20^,^21^. Simultaneous recording of AmB fluorescence lifetime provides insight into molecular organization of the drug in a single lipid bilayer^22^. The experimental model applied has been tested on single giant unilamellar vesicles (GUV) containing popular fluorescence probe Nile blue (NB, Fig. S2).

## Results

### The experimental model

**Figure 1:**
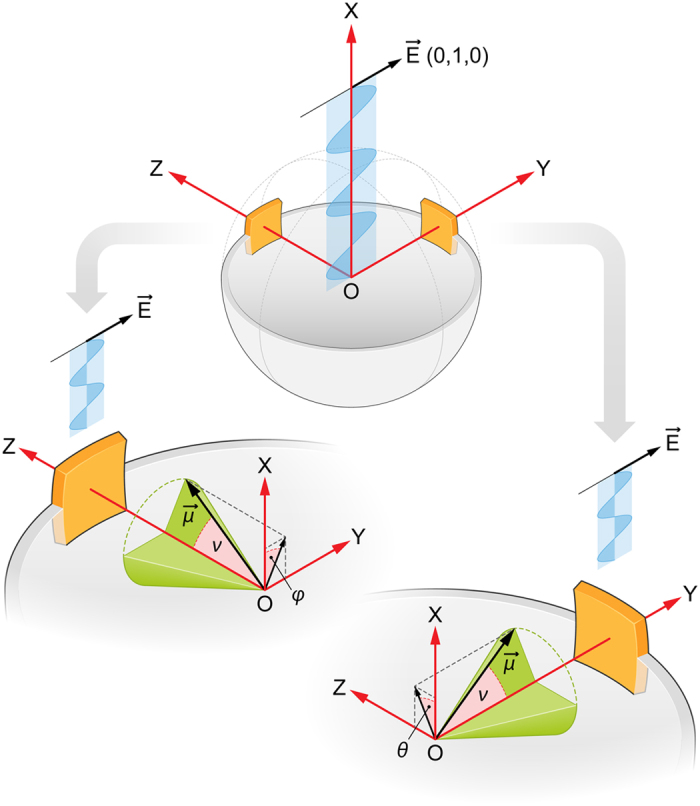
A scheme presenting geometry of analysis of a single lipid vesicle by means of confocal fluorescence microscopy. For explanations see the text.

The idea of the experimental system is presented in Fig. 1. GUV system^23^ has been selected as an experimental model in order to assure relatively low membrane curvature. The centre of the spherical vesicle is located in the centre of the Cartesian laboratory coordinate system. ZOY is the microscope imaging plane and a cross-section plane of a vesicle, characterized by its largest diameter. The electric vector of the scanning laser beam (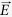) is polarized along the Y axis. Let us consider relatively small fragments of the membrane, one perpendicular to the axis Z and one perpendicular to the axis Y. In our experiment, the virtually planar membrane fragments, 1.5 × 3 μm, have been confined by the focal volume in the direction X (1.468 μm) and by a cone with an aperture angle 20 deg, in the plane ZOY (axis Z ± 10 deg, ~3 μm in the case of a vesicle with the diameter of 17 μm). Presuming that the axis normal to the membrane plane is an axis of symmetry of membrane-bound molecules (homogeneous distribution in the membrane plane) we can introduce the angle ν between the axis normal to the membrane plane and the transition dipole of the molecule of interest (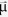). Such an assumption, allows determination of an average cos^2^ν values (<cos^2^ν>) in linear dichroism measurements. In the laboratory coordinates the unit vectors parallel to the transition dipole and to the electric vector of incident light have the following components:




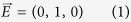







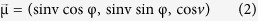




in the membrane fragment spanned by axis Z or:




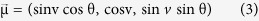




in the membrane fragment spanned by axis Y.

In the equations (2) and (3), φ and θ denote the so-called azimuth angles in the two membrane fragments: φ is the angle between the projection of the dipole transition on the XOY plane and the X axis and θ is the angle between the projection of the dipole transition on the XOZ plane and the X axis (see Fig. 1).

In general, light absorption is proportional to the square of a scalar product of 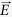 and 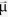 vectors, averaged in the membrane plane (averaging denoted by < >):




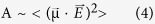




According to this rule, one can calculate separately light absorption in the membrane fragment spanned by the axis Z (A_Z_) and light absorption in the membrane fragment spanned by the axis Y (A_Y_), respectively:




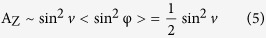







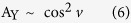




In equation (5), <sin^2^φ> has been replaced by a factor 1/2 due to the homogenous distribution of the transition dipoles with respect to the azimuth angle.

The fluorescence signal recorded from both membrane fragments is proportional to a number of absorbed light quanta:




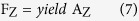







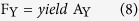




A fluorescence quantum *yield* is proportional to an amplitude-averaged fluorescence lifetime <τ> that was precisely determined for each membrane fragment analysed (α is a proportionality coefficient). Based on this, equations (7) and (8) can be expressed in the following form:




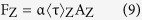







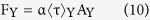




In general, different orientations of molecules with respect to lipid bilayers may be associated with different localization in the membrane and, in consequence, different fluorescence lifetime. In the case of NB, we have found that <τ>_Z_ was identical to <τ>_Y_. This reflects a lack of heterogeneity in localization of NB molecules with respect to the membrane. In order to determine the fluorescence level, emission photons were analysed in two detection channels, with polarization parallel and perpendicular with respect to the direction of the electric vector of the excitation laser beam: F_||_ and F_⊥_ respectively. Total fluorescence level can be expressed as^24^:




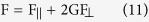




where G denotes the so-called G factor correcting results for inherent polarization of an experimental set-up^24^.

Based on the equations presented above the ratio of the fluorescence signals recorded from both the membrane fragments can be expressed as follow:




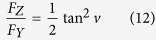




According to this dependency, one can calculate directly an average orientation angle between the direction of the transition dipole of molecules incorporated to a lipid bilayer and the axis normal to the plane of the membrane, based on experimentally determined fluorescence levels in the membrane fragments spanned by the axes Z and Y. In practice, fluorescence signals were integrated from both the symmetric, imaged liposome membrane fragments, located on the both sides of the Y and Z axes.

It has to be noted that the approach developed in this work assumes and is valid in the case in which the directions of dipole transitions of light absorption and fluorescence are the same. Otherwise the angle β between these two directions has to be taken into consideration. This angle can be relatively easily determined from the dependency on fundamental fluorescence anisotropy r_o_^24^:




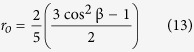




In order to determine r_o_ for NB, fluorescence anisotropy decay kinetics has been recorded and analysed (see Supplementary information Fig. S3). A value of r_o_ = 0.397 determined, lets calculate the angle β = 4.05 deg, which allows to presume that the absorption and emission dipole transitions are close to collinear.

**Figure 2:**
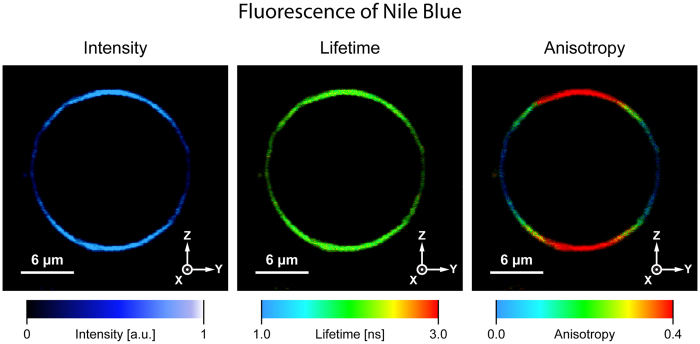
The results of microscopic imaging of a single lipid vesicle containing Nile blue incorporated to the lipid phase. Three panels show fluorescence intensity, lifetime and anisotropy. The images represent a vesicle cross-section in the focal plane of a microscope.

**Figure 3:**
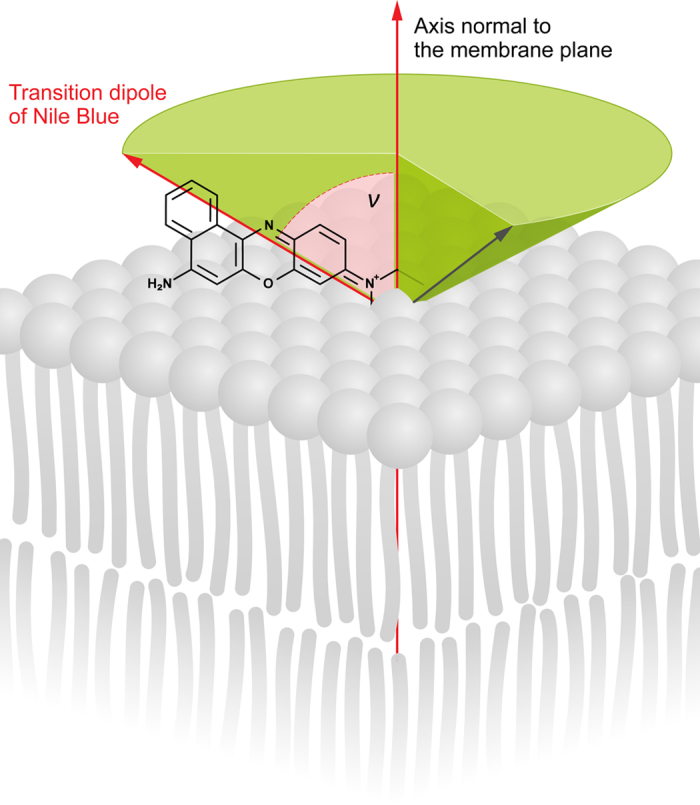
A simplified model presenting orientation of the Nile blue molecule with respect to the lipid bilayer.

The examples of original imaging of GUV containing incorporated NB are presented in Fig. 2. Images represent a cross-section of a scanned vesicle, in the focal plane of a confocal fluorescence microscope. As can be seen, fluorescence intensity is much higher in the lower and upper membrane regions as compared to the regions located at the left- and right-hand sides. Based on photoselection, one can anticipate a relatively flat orientation of the transition dipole of NB with respect to the membrane plane. This can be directly seen from the schematic drawing presenting the idea of photoselection in the experimental system applied (Fig. S4). An orientation of the transition dipole with respect to the membrane plane can also be directly estimated by visual inspection of a liposome image generated based on fluorescence anisotropy (Fig. 2). Owing to the relative immobilization of fluorophores in the lipid membrane environment, fluorescence anisotropy variations are basically determined by a difference between F_||_ and F_⊥_ (see Methods experimental section). This means that in the case of transition dipoles oriented in the membrane plane or more generally, with an orientation angle higher than the magic angle (54.7 deg.), higher fluorescence anisotropy values, coded with red colour, can be expected in the upper and lower sectors of a vesicle. In such a case, the left- and right-hand sectors of the membrane will show lower anisotropy values, coded with blue colour. One can anticipate an inversed distribution of false colours coding fluorescence anisotropy level, in the case of vertical orientation of transition dipoles with respect to the membrane plane. The exact results of determination of angle ν, calculated based on the equation (12), depend slightly on the cone angle used for integration of the fluorescence signal from the membrane fragments spanned by the axes Z and Y, due to the membrane curvature. This can be seen from Table S1 (Supplementary information). Despite certain differences observed, it has to be noted that the approach proposed leads to highly reproducible and precise results (S.D. values below 1 deg.). Based on the results of determination, presented in Table S1, the simplified model of orientation of NB molecules with respect to the membrane plain can be proposed as shown in Fig. 3.

### Amphotericin B in lipid membranes

**Figure 4:**
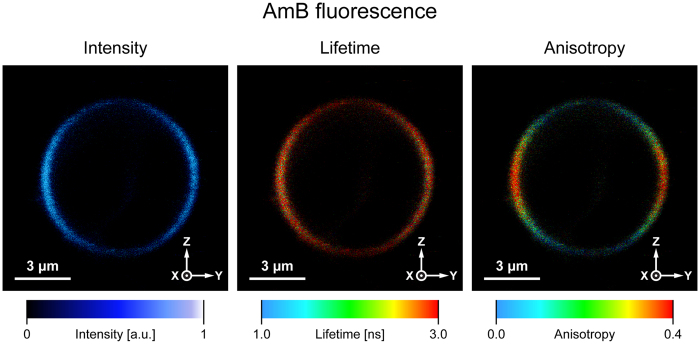
The results of microscopic imaging of a single lipid vesicle containing amphotericin B. Three panels show fluorescence intensity, lifetime and anisotropy. The images represent a vesicle cross-section in the focal plane of a microscope. AmB was present in a concentration of 0.5 mol% in the membrane formed with DPPC containing 10 mol% cholesterol.

**Figure 5:**
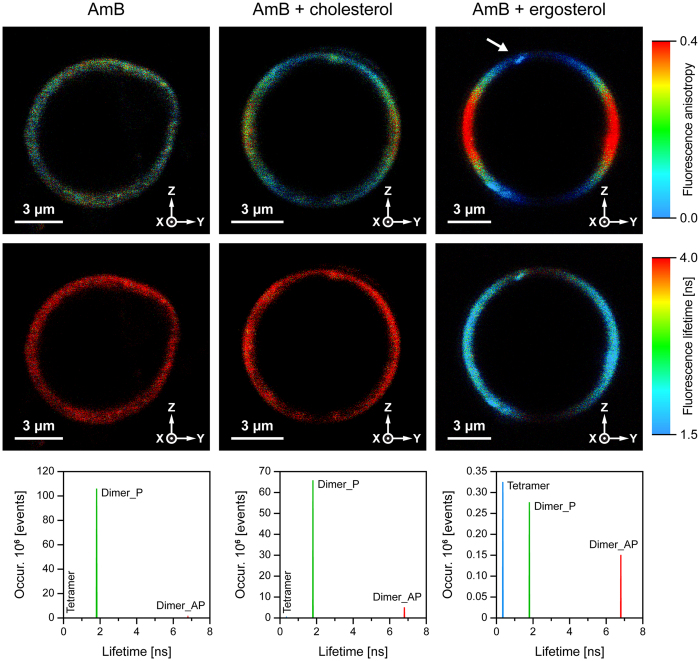
Fluorescence anisotropy and fluorescence lifetime images of unilamellar liposomes containing amphotericin B. Liposomes were formed out of pure lipid (DPPC) and with addition of 10 mol% cholesterol or ergosterol (indicated). Lower panel presents the results of detailed fluorescence lifetime analysis in the images presented. The white arrow points the AmB aggregated structure (discussed in the text).

Figure 4 presents the images of a single GUV, based on fluorescence of AmB incorporated to the lipid membrane. The images presented in Fig. 4 correspond to the images presented in Fig. 2, of a single GUV containing incorporated NB. Figure 5 presents the images based on fluorescence anisotropy and fluorescence lifetimes of AmB incorporated at 0.5 mol% concentration to a single lipid vesicle composed of dipalmitoylphosphatidylcholine (DPPC) and DPPC with addition of 10 mol% of cholesterol (Chol) or ergosterol (Ergo). The results of determination of orientation of the dipole transitions of AmB with respect to the axis normal to the plane of the lipid bilayer are presented in Table 1. Similarly as in the case of NB, the directions of transition dipoles of light absorption and emission by AmB, in both the electronic energy levels S1 and S2 are almost collinear, which is not surprising in the case of a polyene chromophore (S2: β = 4 deg. and S1: β = 6 deg.)^25^. Such a conclusion is also supported by a fundamental anisotropy value of AmB, which has been determined at a level r_o_ = 0.4 in liposomes^26^. As can be seen from Table 1, the transition dipole of AmB in the lipid bilayer system, forms an angle 55.1 ± 2.5 deg. with respect to the axis normal to the plane of the membrane. It has to be noted that the AmB orientation angle determined is practically equal to the magic angle (54.7 deg.), which may indicate a homogeneous distribution of orientation of the drug in such an experimental system. A lack of any preferable orientation can also be directly deduced from the distribution of fluorescence anisotropy in the liposome membranes formed with a pure lipid, in contrast to the lipid membrane containing a fraction of sterols. In the latter case, higher fluorescence anisotropy values, represented by red colour, can be observed in the membrane fragments located in the left- and right-hand sides of the images (spanned by the axis Y, Fig. 5). Such a distribution, opposite to the one observed in the case of NB (Fig. 2), is consistent with a roughly vertical orientation of AmB molecules in the case of the sterol-containing membranes. This effect is particularly pronounced in the case of Ergo- and somewhat less in the case of the Chol-containing lipid bilayer (Fig. 5). An average orientation angle of AmB transition dipole in the membranes modified with Ergo, determined based on equation (12), is 38.0 ± 0.6 deg. The orientation angle is by 10 deg. less than in the case of the Chol-containing lipid bilayer (see Table 1). The orientation angle of the transition dipole of AmB, determined in the case of the membranes containing Ergo, can be interpreted in terms of a vertical orientation of molecules of AmB, taking into account that transition dipole forms an angle of 27 ± 2 deg. with the molecular axis of AmB^25^. It is also possible that a certain small fraction of AmB possesses higher motional freedom, e.g. located in the membrane headgroup region, which makes the average angle as large as 38 deg. Interestingly, such a fraction is definitely higher in the case of the membranes containing Chol, as can be deduced based on the average orientation angle greater by 10 deg. (48.5 deg., see Table 1). The results of the evaluation of orientation of AmB in membranes containing Ergo and Chol, in the present work, qualitatively are in agreement with the data based on the molecular dynamics simulations^27^. Based on the molecular dynamics data, lower orientation angles of AmB, determined for the DPPC+Ergo as compared to the DPPC+Chol system, can be directly related to different orientation of Chol and Ergo in membranes and different ability of both sterols to form complexes with AmB.

**Figure 6:**
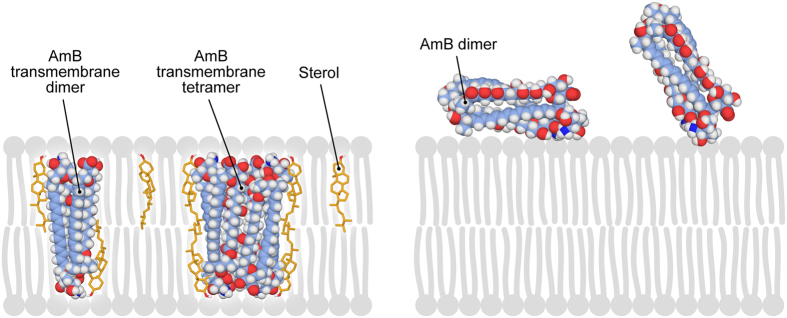
A model presenting localization, molecular organization and orientation of amphotericin B with respect to a lipid membrane with and without sterol molecules. The model membrane on the left-hand side contains ergosterol (yellow).

Analysis of fluorescence lifetime of AmB allows one to gain insight into molecular organization of the drug^22^,^26^. It has been shown that among various molecular organization forms, AmB has a tendency to form antiparallel dimers characterized by a lifetime τ = 6.8 ns, parallel dimers (τ = 1.8 ns), tetramers (τ = 0.35 ns) and to appear as a monomer (τ = 3 ns)^22^. As can be seen, AmB incorporated into the lipid bilayer formed without any sterols appears mostly as a parallel dimer (Fig. 5, Table 2). Such a form has been concluded previously to be localized in the polar headgroup region of the lipid bilayer^22^. As can be seen from Table 2, the fraction of parallel AmB dimers is considerably lower in the sterol-containing membranes (below 30%). In such membranes, the fraction of anti-parallel dimers and tetramers has been additionally detected, which were practically absent in the membranes formed with a pure lipid. Both, the anti-parallel dimers and tetramers of AmB have been concluded to be able to enter a lipid membrane^22^. Interestingly, the fraction of anti-parallel AmB was a dominating one in the Chol-containing membranes. On the other hand, the results of fluorescence lifetime analysis show that the fraction of tetrameric structures of AmB, in the Ergo-containing membranes, is ca. two times higher than in the case of the Chol-containing membranes. At the same time, the average orientation angle of the transition dipole of AmB with respect to the axis normal to the plane of the membrane, is lower in the case of the Ergo-containing membranes than in the case of the Chol-containing membranes (by ca. 10 deg., see Table 1). This suggests that tetrameric forms of AmB are oriented vertically with respect to the membrane (see the model presented in Fig. 6). The conclusion that aggregated structures of AmB, formed in the Ergo-containing membranes and represented by a relatively short fluorescence lifetime, are oriented vertically with respect to the membrane plane has a strong support from the analysis of average fluorescence lifetime in different fragments of a liposome (<τ>_Z_ and <τ>_Y_, see Table S2). Determination of these values is necessary to evaluate orientation of a transition dipole based on equations 9, 10 and 12. Owing to photoselection, vertically-oriented spectral forms contribute to the fluorescence signal in the membrane fragments perpendicular to axis Y (in the left- and right-hand sectors of the membrane, Figs 4 and S4). As can be seen from Table S2, molecular organization forms in these membrane fragments represent distinctly shorter fluorescence lifetimes than the membrane fragments perpendicular to axis Z.

## Discussion

In the present work we developed an experimental approach which enables combined and simultaneous analysis of the molecular organization and orientation of AmB molecules with respect to a single lipid bilayer membrane. The molecular organization is different from those deduced on the basis of the lipid multibilayer system^22^, which seems to be not surprising taking into account that the drug can be localized in the intermembrane compartments of a lipid multi-bilayer^19^. Such a conclusion can be drawn based on comparison of the parameters representing AmB-containing lipid membrane thickness, determined based on a small-angle neutron scattering in a suspension of small unilamellar vesicles and X-ray diffraction recorded in a lipid multi-bilayer system^19^. Importantly, sensitivity of the technique applied in the present work, enables analysis of relatively low concentrations of the drug with respect to lipids (0.5 mol%). Such low concentrations are relevant from the standpoint of AmB interactions with natural membranes, under clinical conditions. The results show that a parallel dimer of AmB, which is formed with high efficiency in a broad range of experimental systems^22^,^28^, is also a major molecular organization form of the drug in a lipid membrane. Relatively low fluorescence anisotropy of this structure reflects its high motional freedom and therefore implies localization in the surface region of the polar headgroup zone. Interestingly, the presence of sterols in the lipid phase results in formation of other AmB structures, antiparallel dimers and tetramers (Fig. 5, Table 2). Such structures, have been concluded previously to be preferentially formed in the hydrophobic membrane core^22^. The more vertical orientation of AmB molecules, in the sterol-containing membranes, corroborates with such a conclusion. Importantly, the effect of sterols in reorientation of AmB to vertical with respect to the membrane plane, combined with the membrane penetration by molecules of the drug, have been very recently concluded on the basis of the molecular dynamics simulations^29^. It also has to be mentioned, that very similar effect of sterols has been observed in the case of another polyene antibiotic, filipin^17^. The fact that Ergo, a sterol present in biomembranes of fungi, is more effective than Chol in providing conditions for AmB molecules to be located inside a lipid bilayer and to form aggregated structures, reported in the present work, underlies most probably a molecular selectivity of the drug in its pharmacological action against fungi. As can be seen (Fig. 5, Table 2), AmB can form tetrameric structures, in the Ergo-containing lipid membranes. An effective abundance of such structures in the Ergo-containing lipid membranes may be even higher than it can be deduced from relative fluorescence lifetime amplitudes (see Table 2), owing to the fact that spectral forms characterized by shorter fluorescence lifetimes display usually lower fluorescence quantum yield. Tetrameric structures of AmB have been proposed to be able to act as transmembrane ion channels, affecting physiological ion transport^22^,^26^. The conclusion that such a transmembrane pore can be formed by association of AmB dimeric structures and does not need to be formed via assembly of two semi-channels, each one present in a single lipid monolayer constituting a membrane, has also very strong support from the results of the studies carried out with application of solid-state NMR spectroscopy^30^ and electrophysiological techniques^31^. On the other hand, any presence of AmB in the lipid phase is associated with serious destabilization of the membrane structure^32^ and can cause ion leaking^33^. Such direct presence of AmB in the lipid phase is also associated with the presence of Chol (Fig. 5, Table 2), which can explain severe side effects of the drug to patients. In the present study, we have not observed any extramembraneous, sponge-like molecular aggregates of AmB in the sterol-containing systems. During our preparation we tried to eliminate large aggregates of AmB, at the initial stages of AmB purification and sample preparation, in order to provide freedom for formation of molecular assemblies of the drug in a lipid phase. We have also applied relatively low concentration of AmB with respect to lipids (0.5 mol%). Additionally, aggregated forms of AmB have usually lower fluorescence quantum yield and owing to this fact presence of AmB aggregates may be underestimated. Despite that we observed some aggregated structures of fluorophores, visible on liposome images, in particular within the sterol-containing DPPC membranes. Such structures are manifested as regions characterized by enhanced fluorescence intensity and distinctively different fluorescence anisotropy and fluorescence lifetimes. The fact that fluorescence intensity detected in such aggregated structures of AmB is relatively high suggests that direct contact between fluorophores is hindered by interaction with molecules of a sterol. One of such structures is pointed by the white arrow in Fig. 5. It is therefore possible, that one of the modes of action of AmB in biomembranes is associated with a sequestration of sterol molecules from the lipid phase and immobilization them within two-component, sterol-AmB aggregated molecular ensembles^10^.

From analysis of the literature data and the results presented in this work it follows that effect of AmB on lipid membranes is multi-modal. Among various molecular mechanisms associated with interaction of AmB with lipid membranes are destabilization of molecular order of lipids (e.g. demonstrated by decreased cooperativity of the main phase transition^32^) and self-assembly of the drug molecules within the lipid phase^26^,^31^,^34^,^35^. The first mechanism can be responsible for nonspecific negative effects on the structural and dynamic membrane properties. On the other hand, the second mechanism can be associated with formation of transmembrane pores, disturbing significantly transmembrane ion transport^8^,^9^,^33^,^36^,^37^,^38^,^39^. Important for the biological activity of AmB is dependence of both mechanisms on presence of sterols in the membrane lipid phase. The results of both theoretical and experimental studies show that interaction of AmB to Ergo is stronger than to Chol^27^,^31^,^32^,^34^,^40^. Such a difference could be considered as directly responsible for a certain selectivity of AmB towards biomembranes of fungi, containing Ergo. On the other hand, interaction to Chol, nonspecific binding to lipid membranes or to other biomolecules is most probably associated with a pronounced toxicity to patients. One can ask, whether it is at all possible to separate pharmacologically relevant biological activity of AmB from toxic side effects of the drug? According to the results presented in this work, the sterol-containing membranes provide favourable conditions for AmB to enter the lipid phase (i), adopt a vertical orientation (ii) and to form molecular assemblies (iii) which can potentially disturb physiological ion equilibrium of the cell. Recently, we have reported a synthesis of AmB-silver hybrid nanoparticles in which molecules of the drug, immobilized at the metal surface, had exposed their large portion to interaction to other molecules, e.g. sterols^41^. The fact that AmB present in such structures is not able to form molecular aggregates is most probably responsible for relatively low cytotoxicity of the drug, as compared to other formulations^41^. On the other hand, AmB in hybrid nanoparticles has shown enhanced antimycotic activity^41^. It is therefore possible that this effect is associated with sequestration of Ergo from the membranes, since the Raman microscopy imaging demonstrated direct interaction of AmB-silver nanostructures with a hypha of *Aspergillus niger*^41^. Further analysis of the effect of AmB on natural biomembranes, by application of different experimental techniques, including the approach developed and described in the present work, shall bring more complete view on a possibility of minimization of toxic side effects of AmB combined with enhanced pharmacological effectiveness against fungal infections.

## Methods

### Chemicals

Nile blue (NB) was purchased from Carl Roth GmbH (Germany), antibiotic amphotericin B (AmB), L-α-dipalmitoylphosphatidylcholine (DPPC), cholesterol (Chol) and ergosterol (Ergo) were purchased from Sigma Aldrich (USA). All other chemicals used in the preparations were of analytical grade. In order to assure high purity of AmB for spectroscopic measurements, the drug was purified chromatographically, directly before use, according to a procedure described previously^22^,^26^.

### Liposome preparation

Giant Unilamellar Vesicles (GUV) were formed of DPPC or DPPC with either cholesterol or ergosterol at 10 mol% with respect to lipid. We have selected a relatively low concentration of sterols (10 mol% with respect to lipid) in order to analyze a specific interaction of AmB with ergosterol and cholesterol, not covered by the effects of sterols on a membrane fluidity. Final lipid concentration in a water phase was 27 mM. In order to prepare liposomes containing NB, the pigment was added to a lipid solution in ethanol, at concentration 0.05 mol% with respect to DPPC. In order to prepare liposomes containing AmB, a solution of the antibiotic prepared in DMSO (to assure monomeric state of molecules of a drug) was admixed to an ethanolic solution of DPPC. AmB concentration was 0.5 mol% with respect to lipid. Constituents of a lipid phase of liposomes were deposited to two platinum electrodes (35 × 4 × 0.5 mm) by means of evaporation from a solution, under a stream of gaseous nitrogen. Residuals of organic solvents were removed during incubation for 1 h under vacuum. The two platinum electrodes, covered by deposited lipid films, were fixed in the Teflon holder at a distance of 5 mm. Electrodes were placed in a cuvette containing buffer solution (1.4 mL, 20 mM Tricine, 10 mM KCl, pH 7.4). Electric connections were attached to an AC field supply (DF 1641A). GUV electroformation was carried out over 2 h with an applied AC sinusoidal field with 10 Hz frequency and voltage 3 V (peak-to-peak), according to the recommendations from the literature^42^. The temperature during electroformation was stabilized at 50 °C (above the main phase transition of membranes formed with DPPC, ~41 °C).

### Spectroscopic measurements

Fluorescence emission and excitation spectra were recorded with application of a FS5 fluorescence spectrophotometer (Edinburgh Instruments, U.K.). Fluorescence spectra were corrected for the photomultiplier sensitivity and for the lamp spectrum. Spectral widths for fluorescence excitation and emission were set at 5 nm and 3 nm, respectively in the case of the emission spectra and at 3 nm and 5 nm in the case of excitation spectra.

### Microscopy measurements

The experiments were performed on a two-channel MicroTime 200 (Picoquant, Germany) confocal system coupled to Olympus IX71 inverted microscope. The instrument was equipped with the objective piezo-scanner with 80 μm × 80 μm imaging range at nominal 1 nm positioning accuracy. A sample was illuminated with 635 nm pulsed laser, in the case of liposomes containing NB, and with 405 nm pulsed laser, in the case of liposomes containing AmB, focused on interesting objects by water immersed objective (Olympus Plan Apo NA = 1.2, 60×). The observation was made using dichroic (ZT 523/640 RPC from Chroma-AHF Analysentechik) and 650 nm long wavelength pass filter (BLP01-635-25 from Semrock), in the case of 635 nm laser and with ZT 405RDC dichroic and HQ 430lp filter, both from Chroma-AHF Analysentechnik, in the case of 405 nm laser. The confocal pinhole of 50 μm in diameter was used. Fluorescence beam was split by polarizing cube and observed by two orthogonally polarized analysers (Single Photon Avalanche Diodes). The perpendicular (F_⊥_) and parallel (F_||_) intensities were measured and further used to calculate the anisotropy as defined:




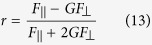




Polarization directions are referred to polarization of excitation laser beam. G was an instrumental correction factor (e.g. 1.044). A value of factor G was determined before each experiment, in separate measurements carried out with a long-lifetime fluorescence probe.

The effective confocal volume V_conf_ of the system was calculated based on the formula referring to its ellipsoidal shape approximation:




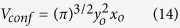




where y_o_ is a lateral and x_o_ the axial radius of the volume. In our configuration alignment y_o_ = 140 nm and x_o_ = 734 nm, for a 405 nm laser. Hence, V_conf_ = 0.08 fL.

The microscopy system recorded fluorescence anisotropies and lifetimes of the sample simultaneously. The intensity decays were analyzed in terms of an exponential model using SymPhoTime 64 v. 2.1 software (PicoQuant).

### Fluorescence anisotropy decay measurements

Time-resolved fluorescence anisotropy data for NB ethanolic solution were recorded with application of FT-300 time-resolved fluorescence spectrometer (Picoquant, Germany), with a laser and filter combination the same as in case of microscopy measurements. The observation was made after monochromator set at fluorophore emission maximum (671 nm, see Fig. S2). Two emission intensity decays were recorded: F_⊥_(t) and F_||_(t). From these decays, the anisotropy as a function of time was determined. The anisotropy decay data were satisfactory fitted (χ^2^(reduced) = 1.3) with one-exponential function:




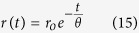




where r_o_ is an initial anisotropy contribution and θ is a fluorophore rotational correlation time (see Fig. S3).

## Additional Information

**How to cite this article**: Grudzinski, W. *et al*. Molecular organization, localization and orientation of antifungal antibiotic amphotericin B in a single lipid bilayer. *Sci. Rep*. **6**, 32780; doi: 10.1038/srep32780 (2016).

## Supplementary Material

Supplementary Information

## Figures and Tables

**Table 1:** Results of determination of orientation angle ν of AmB with respect to the axis normal to the plane of the lipid bilayer, determined based on equation (12).

Membrane composition	DPPC	DPPC + Chol	DPPC + Ergo
Orientation angle ν [deg]	55.1 ± 2.5	48.5 ± 0.9	38.0 ± 0.6

Membranes were formed with DPPC with and without cholesterol and ergosterol present at 10 mol%. AmB was incorporated to liposomes at 0.5 mol% with respect to lipid. Data represent arithmetic mean ± S.D. from 5 experiments.

**Table 2:** Molecular organization forms of AmB in lipid membranes formed with DPPC without and with Chol and Ergo, present in the lipid phase at 10 mol%.

Organization form of AmB	Fraction [%]		
	DPPC	DPPC + Chol	DPPC + Ergo
Tetramer(τ = 0.35 ns)	0.2 ± 0.1	17.6 ± 2.4	39.6 ± 7.4
Dimer parallel(τ = 1.8 ns)	99.0 ± 0.4	29.0 ± 2.6	27.3 ± 6.5
Dimer anti-parallel(τ = 6.8 ns)	0.8 ± 0.3	53.4 ± 1.2	33.1 ± 11.8

AmB was incorporated to liposomes at concentration 0.5 mol% with respect to lipid. Data represent arithmetic mean ± S.D. from 5 experiments.
