# Supplementary material for: Molecular organization, localization and orientation of antifungal antibiotic amphotericin B in a single lipid bilayer
Source: Sci Rep. 2016 Sep 13;6:32780. doi: 10.1038/srep32780 (PMC5020354; doi:10.1038/srep32780)
Supplement: Supplementary Information [file srep32780-s1.pdf]

Supplementary Information

Molecular organization, localization and orientation of antifungal antibiotic amphotericin B in a single lipid bilayer

Wojciech Grudzinski, Joanna Sagan, Renata Welc, Rafal Luchowski, Wieslaw I. Gruszecki

Department of Biophysics, Institute of Physics, Maria Curie-Skłodowska University, Lublin, Poland

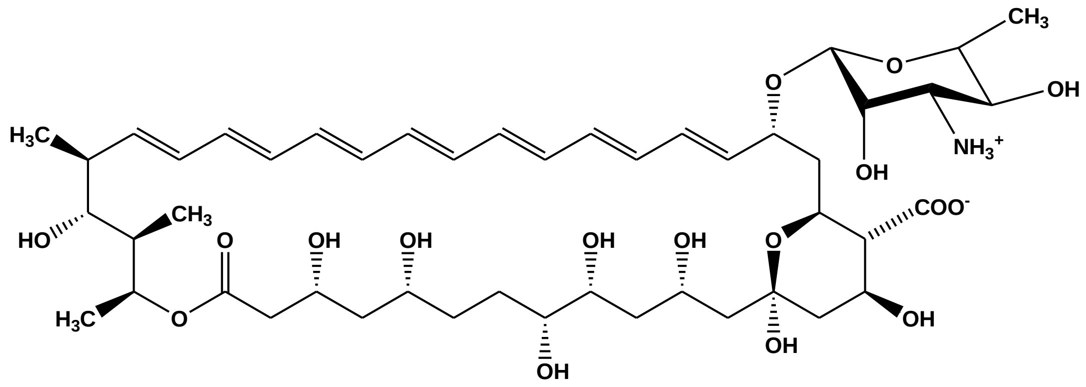

Figure S1 Chemical structure of amphotericin B molecule.

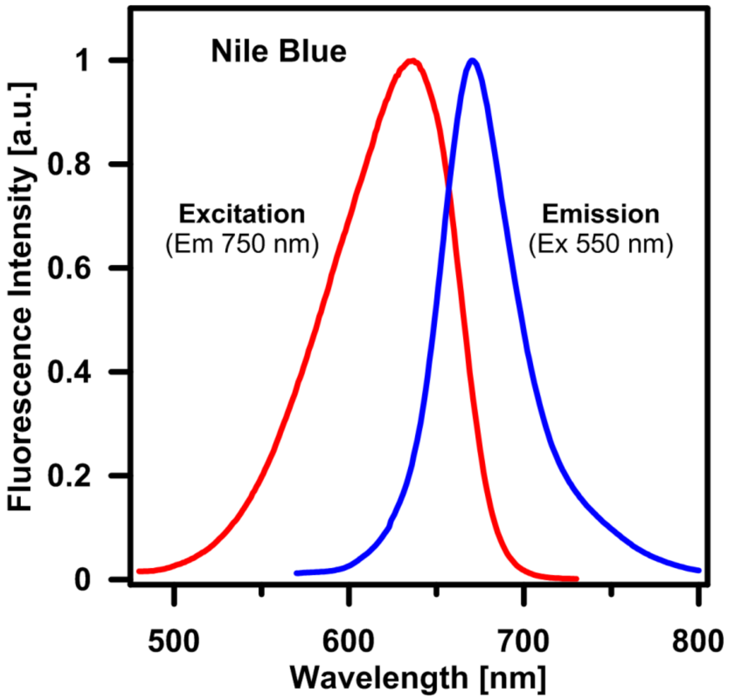

Figure S2 Comparison of fluorescence excitation and fluorescence emission spectra of Nile blue. The fluorescence excitation (red) and emission (blue) spectra were normalized at maximum. The spectra were recorded from a solution in ethanol. Relatively small shift of the spectra, observed, suggests that fluorescence emission originates from the same electronic energy state to which molecule is excited by light absorption.

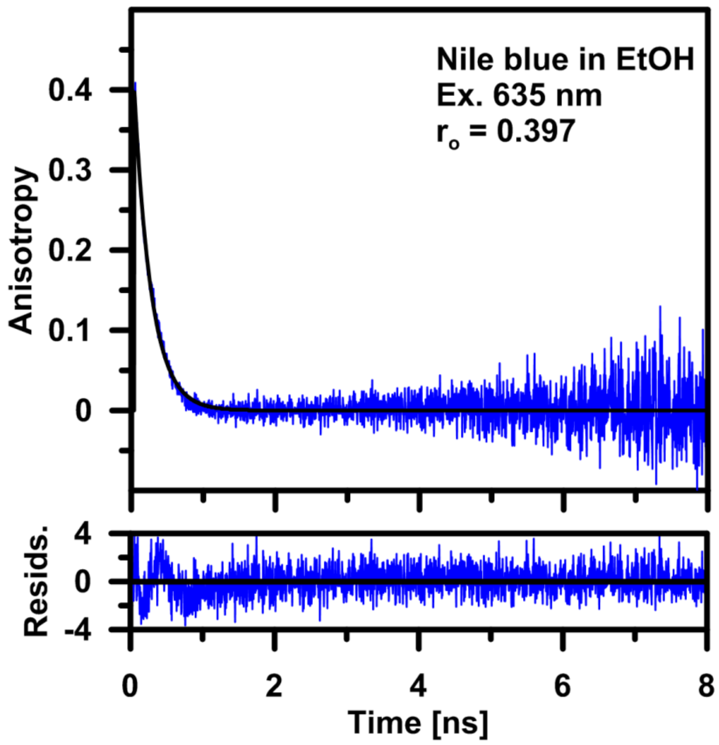

Figure S3 Fluorescence anisotropy decay kinetics of Nile blue in ethanolic solution. Value of fundamental anisotropy r_o was determined by fitting the experimental dependency with an exponential function.

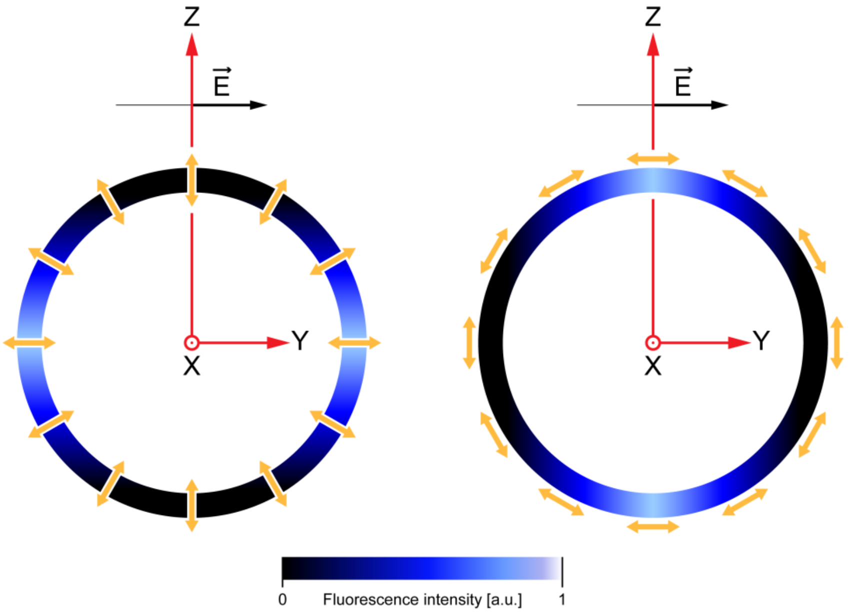

Figure S4. Schematic drawing showing difference in fluorescence intensity, due to photoselection, of molecules oriented perpendicular and parallel with respect to the membrane plain.

Table S1. Comparison of values of an orientation angle ν of the transition dipole of Nile blue with respect to the axis normal to the membrane plane, determined based on equation (12,) on geometrical parameters of fluorescence integration. Data represent arithmetic mean ± S.D. from 5 experiments.

Integration cone angle [deg]	60	40	20
<ν> [deg]	60.9	61.7	62.1
S.D. [deg]	0.2	0.3	0.9

Table S2. Amplitude-averaged fluorescence lifetime of amphotericin B incorporated to liposomes formed with DPPC and DPPC with Chol or Ergo. Sterols was present in the membranes at concentration 10 mol%, AmB at concentration 0.5 mol%. Fluorescence lifetime was determined in the membrane fragments perpendicular to axis Y and axis Z, according to equations 9 and 10. Data represent arithmetic mean from 5 experiments ± S.D.

Membrane composition	DPPC	DPPC + Chol	DPPC + Ergo
$\langle\tau\rangle_Y$ [ns]	1.853 ± 0.001	1.461 ± 0.218	0.595 ± 0.045
$\langle\tau\rangle_Z$ [ns]	1.841 ± 0.035	1.618 ± 0.198	0.901 ± 0.031
